# 血小板淋巴细胞比值作为非小细胞肺癌预后因素的*meta*分析

**DOI:** 10.3779/j.issn.1009-3419.2019.05.05

**Published:** 2019-05-20

**Authors:** 浩然 陈, 昊 薛, 文静 刘, 方方 吴, 一托 王, 红军 高

**Affiliations:** 1 100850 北京，军事科学院军事医学研究院研究生部 Academy of Military Medical Sciences, 100850 Beijing, China; 2 100071 北京，解放军总医院第五医学中心肺部肿瘤内科 Department of Lung Neoplasms, Fifth Medical Center of the Chinese PLA General Hospital, Beijing 100071, China; 3 100700 北京，解放军总医院第七医学中心放射科 Department of Radiology, Seventh Medical Center of the Chinese PLA General Hospital, Beijing 100700, China

**Keywords:** 肺肿瘤, 血小板与淋巴细胞比值, *Meta*分析, 总生存期, 无病生存期, Lung neoplasms, Platelet to lymphocyte ratio, *Meta*-analysis, Overall survival, Disease-free survival

## Abstract

**背景与目的:**

目前研究显示，血小板淋巴细胞比值（platelet lymphocyte ratio, PLR）在肾细胞癌、食管癌、胃癌、肝癌以及结肠癌中均有重要的预后价值。本研究旨在通过*meta*分析，评估PLR对非小细胞肺癌（non-small cell lung cancer, NSCLC）患者的预后价值。

**方法:**

使用计算机电子系统对PubMed、EMBASE、Web of Science、Medline、Cochrane Library、中国知网、中国生物医学文献数据库、维普、万方数据库进行系统文献检索，查阅研究PLR与总生存期（overall survival, OS）和无病生存期（disease-free survival, DFS）之间关联的文献。提取每个符合条件的研究数据，使用风险比率（hazard risk, HR）和95%置信区间（95% confidence interval, 95%CI）进行系统评价分析以评估PLR的预后价值，检索时限为建库至2018年11月。

**结果:**

我们共纳入15篇研究文献涉及5524名患者进行系统评价分析。*Meta*分析结果显示：较高PLR组的OS明显低于较低PLR组的OS（HR=1.69, 95%CI: 1.45-1.97, *P* < 0.000, 01, *I*^2^=46.2%, *P*异质性=0.026）；较高PLR组的DFS明显低于较低PLR组的DFS（HR=1.41, 95%CI: 1.14-1.74, *P*=0.001, *I*^2^=46.2%, *P*异质性=0.026）。亚组分析显示，按种族、样本大小、PLR临界值和治疗方法分组后，PLR较高组的OS仍明显低于PLR较低组（*P* < 0.05）。

**结论:**

PLR增高与NSCLC患者预后不良有关，因此PLR可能作为NSCLC患者重要的生物预测标记，但其临床应用仍需要未来通过更多的研究来验证。

## Abstract (en)

**Background and objective:**

Current research shows that platelet to lymphocyte ratio (PLR) has important prognostic value in renal cell carcinoma, esophageal cancer, gastric cancer, liver cancer and colon cancer. The aim of the study is to evaluate the prognostic value of PLR in non-small cell lung cancer (NSCLC) through *meta*-analysis.

**Methods:**

Literature search for PubMed, EMBASE, Web of Science, Medline, Cochrane Library, China National Knowledge Internet (CNKI), China Biomedical Medicine disc (CBMdisc), VIP, Wanfang Database using computer electronic system to study the association between PLR and overall survival (OS) and disease-free survival (DFS). Each eligible study data is extracted and a *meta*-analysis is performed using the hazard risk (HR) and 95% confidence interval (95%CI) to assess the prognostic value of PLR, the time limit for the search is to build the library until November 2018.

**Results:**

We include a total of 15 research literatures involving 5, 524 patients for *meta*-analysis. According to the results of the *meta*-analysis: The OS of the higher PLR group is significantly lower than that of the lower PLR group (HR=1.69, 95%CI: 1.45-1.97, *P* < 0.000, 01, *I*^2^=46.2%, P_heterogeneity_=0.026); the DFS of the higher PLR group is significantly lower than that of the lower PLR group (HR=1.41, 95%CI: 1.14-1.74, *P*=0.001, *I*^2^=46.2%, P_heterogeneity_=0.026). Subgroup analysis show that the OS of the higher PLR group is still significantly lower than the lower PLR group (*P* < 0.05) after grouping by ethnicity, sample size, PLR cutoff value and treatment.

**Conclusion:**

Increased PLR is associated with poor prognosis in NSCLC, so PLR may be an important biological predictive marker for NSCLC patients, however, its clinical application still needs to be verified through more research in the future.

肺癌是引起癌症相关死亡最常见的病因，它的5年生存率仅约16%^[1]^。在肺癌的诊断中，约80%为非小细胞肺癌（non-small cell lung cancer, NSCLC），虽然目前多种治疗对于NSCLC患者的预后有了明显的改善，但现状仍不容乐观。改善长期生存的一个重要突破点就是应用准确的生物预测标志物，从而指导相关治疗策略并监测疾病的进展。因此，准确的生物预测标志物对于NSCLC患者的预后至关重要。为了更好的评估预后，已研究出许多与预后有关的因素，如肿瘤-淋巴结-转移（tumor node metastasis, TNM）分期、患者状态、体重变化等。近几年，许多专家认为全身炎症反应在癌症的发生和发展中起着非常重要作用^[2]^。在生物学途径中，细胞因子、趋化因子以及炎症介质的增多能够促进血管生成、造成局部免疫的抑制以及抑制细胞凋亡，而且能够增加肿瘤的转移风险^[3]^。目前已有许多研究显示，血小板、淋巴细胞、中性粒细胞、C-反应蛋白（C reactive protein, CRP）以及格拉斯哥预后评分（Glasgow Prognostic Score, GPS），它们在多种癌症中都具有重要的预后价值^[4-5]^。那么血小板淋巴细胞比值（platelet lymphocyte ratio, PLR）对于癌症是否有重要的预后价值就需要进一步分析。综合已发表的数据，PLR在肾细胞癌、食管癌、胃癌、肝癌以及结肠癌中均有重要的预后价值^[6-10]^。但PLR在NSCLC中的预后价值仍有争议^[11-13]^。因此本研究旨在利用*meta*分析来评估PLR对于NSCLC患者的预后价值。

## 1 资料和方法

### 1.1 文献检索

该*Meta*分析是根据系统综述和*Meta*分析优先报告的条目（Preferred Reporting Items for Systematic Reviews and *Meta*-Analyses, PRISMA）为指南进行开展的^[14]^。使用计算机电子系统对PubMed、EMBASE、Web of Science、Medline、Cochrane Library、中国知网、中国生物医学文献数据库、维普、万方数据库进行系统文献检索，以评估PLR对于NSCLC患者的预后价值。检索策略为以下专业术语的组合：英文检索关键词：（platelet-to-lymphocyte ratio or platelet lymphocyte ratio or platelet lymphocyte ratio or PLR）and（lung cancer or lung carcinoma or non-small cell lung cancer or non small cell lung cancer or NSCLC）；中文检索关键词：（血小板与淋巴细胞比率或血小板淋巴细胞比率）和（肺癌或肺恶性肿瘤或NSCLC）；检索时限截至2018年11月，没有相关文献发表的时间以及区域的限制。检索的文献中仅对包含人体的研究进行*meta*分析，此外也检索纳入合格文献中的参考文献，以防相关文献的漏检。

### 1.2 纳入标准

如果文献符合以下所有标准，则将其纳入到*meta*分析中：①经过病理学确诊为NSCLC的患者；②治疗前测定PLR；③研究目的为PLR与疾病预后[（总生存期（overall survival, OS）和无病生存期（disease-free survival, DFS）]之间的关系；④相关文献中报告了风险比率（hazard risk, HR）和95%置信区间（95% confidence interval, 95%CI）；⑤相关文献语言为英文或中文。

### 1.3 排除标准

① 摘要、病例报告、综述、信件、评论文章和会议报告；②相关文献缺少合格的研究数据。

### 1.4 数据提取

由两位评价员独立评估和提取相关研究的数据，对所有研究进行了双重检查，如有分歧则通过讨论和共识进行解决，提取的相关信息包括以下内容：①纳入文献的详细信息：包括作者、国家、发表年份以及研究起止时间等；②研究人群的特征：包括样本大小、疾病分期、治疗手段和PLR临界值；③PLR与疾病预后（OS和DFS）之间关联的HR和95%CI，如果在同一篇文章中提出了多个估计值，则选择最具有代表意义的一个（多变量分析优于单变量分析）。

### 1.5 质量评价

所有纳入的相关文献均通过纽卡斯尔-渥太华量表（The Newcastle-Ottawa Scale, NOS）进行质量评价^[15]^，该量表评价标准包括：对研究人群的选择：暴露组的代表性如何、如何选择非暴露组、用何方法确定暴露因素、研究开始时暂无需要观察的结局指标（0分-4分）；组间的可比性：当设计和分析时考虑暴露组和非暴露组的可比性（0分-2分）；结果评价：该研究对于结果的评价是否充分、发生结果后的随访时间是否足够长、暴露组和非暴露组的随访时间是否充分（0分-3分）。其得分范围为0分-9分，其中≥7分为高质量研究（表 1）。经过评分后如 < 7分则排除。王一托和薛昊分别为我院的博士研究生和硕士研究生，评分期间如两位评价员产生分歧，通过讨论解决，从而达成共识。

**1:** 所有纳入文献相关信息 All relevant information in the literature

Literature	Published time	Country	Ethnicity	Study period	Study design	Sample size(male/female)	TNM classification	Treatment	PLR cutoff value	Survival analysis	HR	95%CI	NOS score
Sánchez-Lara *et al* ^[17]^	2012	Mexico	Caucasian	2009-2011	Prospective	119 (55/64)		C	150	OS	1.16	0.52-2.50	7
Liu *et al* ^[18]^	2013	China	Asian	2001-2012	Retrospective	210 (139/71)	Ⅲ-Ⅳ	C	152.6	OS	2.025	1.405-2.919	8
Unal *et al* ^[19]^	2013	Turkey	Caucasian	NA	NA	94 (88/6)	Ⅱ-Ⅲ	C+R	194	OS	1.87	1.20-2.91	8
										DFS	1.6	1.03-2.49	
Pinato *et al* ^[20]^	2014	UK	Caucasian	2004-2011	Prospective	220 (110/110)	Ⅰ-Ⅲa	S+C/R	300	OS	1.6	0.6-5.6	7
Zhang *et al* ^[21]^	2014	China	Asian	2006-2009	Retrospective	400 (272/128)	Ⅰ-Ⅱ	S	171	OS	1.985	1.269-3.104	8
										DFS	1.534	1.022-2.304	
Wu *et al* ^[12]^	2014	China	Asian	2007-2012	Retrospective	366 (246/120)	Ⅲ-Ⅳ	C	119.5	OS	1.189	0.852-1.658	7
Kos *et al* ^[11]^	2015	Turkey	Caucasian	2005-2011	Retrospective	145 (130/15)	Ⅰ-Ⅳ	S/C/R	198.2	OS	1.45	1.04-2.32	7
										DFS	1.079	0.729-1.596	
Kawashima *et al* ^[22]^	2015	Japan	Asian	1998-2012	Retrospective	1, 043 (671/372)	Ⅰ-Ⅲ	S	300	OS	2.35	1.45-3.82	7
Zhang *et al* ^[23]^	2015	China	Asian	2004-2008	Retrospective	678 (449/229)	Ⅰ-Ⅲ	S	106	OS	1.298	1.035-1.629	7
Kim *et al* ^[24]^	2016	Korea	Asian	2002-2007	Retrospective	202 (169/33)	Ⅰ-Ⅲ	S+R+C	160	OS	3.473	1.765-6.835	8
Diem *et al* ^[25]^	2017	Switzerland	Caucasian	2015-2016	Retrospective	52 (29/23)	Ⅲ-Ⅳ	C	262	OS	3.32	1.66-6.55	8
Yuan *et al*^[26]^	2017	China	Asian	2005-2009	Retrospective	1, 466 (1, 058/408)	Ⅰ-Ⅲa	S+C	204	OS	1.406	1.126-1.755	8
Toda *et al*^[27]^	2017	Japan	Asian	2008-2012	Retrospective	327 (213/114)	Ⅰ-Ⅲ	S+C	162	OS	1.9	1.2-3.2	7
Wang *et al*^[28]^	2017	China	Asian	2005-2013	Retrospective	134 (81/53)	Ⅰ-Ⅲa	S	145	OS	2.005	1.294-3.106	7
										DFS	1.609	1.004-2.577	
Yi *et al*^[29]^	2018	China	Asian	2008-2015	Retrospective	68 (50/18)	Ⅲ-Ⅳ	C	130	OS	1.073	0.540-2.133	8
NA: not available; C: chemotherapy; R: radiotherapy; S: surgery.													

### 1.6 统计及分析方法

汇总每项研究中的HR及95%CI，评估PLR对于NSCLC患者的预后价值。我们使用*Cochran’s Q*统计量检验法，然后根据*I*^2^值和*P*值分析不同研究间的异质性。在Cochran Handbook的5.0及以上版本中根据*I*^2^值大小将异质性分为4个程度：轻度异质性0%-40%，中度异质性40%-60%，较大异质性50%-90%，很大异质性75%-100%^[15]^。在Cochrane系统评价中只要*I*^2^≤50%，*P* > 0.10时则其异质性就可接受^[16]^。当纳入的不同研究没有异质性时，则使用固定效应模型合并，并生成得出HR及95%CI；否则则使用随机效应模型，并进行亚组分析及*meta*回归讨论异质性来源。当合并得出的HR > 1时则表明生存率较差，如果95%CI不包含1，则认为其具有统计学意义。还需进行灵敏度分析以评估结果是否稳定，同时使用*Begg*法和*Egger*法生成的漏斗图评估相关研究的发表偏倚，必要时采用*Begg*法和*Egger*法对发表偏倚进行定量分析。所有的统计检验均为双侧，显着性水平*α*设定为0.05，当*P* < 0.05时有统计学意义。纳入的所有研究分析均使用统计软件Revman 5.3及Stata 12.0。

## 2 结果

### 2.1 文献检索

根据初始检索策略总共检索到了832篇研究文献，根据纳入排除标准筛选、排除重复研究并筛选标题或者摘要后，得到58篇关于PLR和NSCLC预后有关的研究文献，经过进一步评估，排除39篇未提供特定数据的文献以及NOS评分 < 7分的4篇文献（山长平等、Cannon等、Lan等、Luo等）。最终纳入了15篇符合条件的研究文献，共计5, 524例NSCLC患者，研究纳入的流程图见图 1。

**1:**
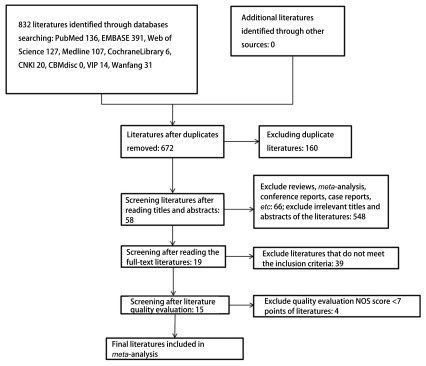
*Meta*分析的研究纳入流程图 Flow diagram of study selection for the *meta*-analysis

### 2.2 纳入文献的特点

纳入的15篇文献中有8项研究（Pinato等^[20]^、Zhang等^[21]^、Kawashima等^[22]^、Zhang等^[23]^、Kim等^[24]^、Yuan等^[26]^、Toda等^[27]^、Wang等^[28]^）包括可以手术治疗的病例（Ⅰ期-Ⅱ期或Ⅰ期-Ⅲ期），有6项研究（Sánchezlara等^[17]^、Liu等^[18]^、Unal等^[19]^、Wu等^[12]^、Diem等^[25]^、Yi等^[29]^）仅含有不能手术的病例（Ⅲ期-Ⅳ期），有1项研究（Kos等^[11]^）则涵盖了所有阶段病例（Ⅰ期-Ⅳ期）。此篇*meta*分析共纳入5, 524例NSCLC的患者（3, 760例男性，1, 764例女性）。每项研究中患者的数量范围为52例-1, 466例，其中位数为202，使用的PLR的临界值范围为106-300，其中位数为162。纳入的文献的主要特征见表 1。

### 2.3 *Meta*分析结论

综合纳入的文献可见，有15项研究都提供了有关PLR及OS的数据，其中4项研究提供了PLR及DFS的数据。根据我们的汇总结果表明：PLR增高与OS（HR=1.69, 95%CI: 1.45-1.97, *P* < 0.000, 01, *I*^2^=46.2%, *P*异质性=0.026）（图 2）和DFS（HR=1.41, 95%CI: 1.14-1.74, *P*=0.001, *I*^2^=0%, *P*异质性=0.462）（图 3）的降低密切相关。通过对*I*^2^值与*P*异质性值进行分析，纳入与NSCLC患者DFS相关的文献无明显异质性，但是纳入与NSCLC患者OS相关的研究文献之间存在中等程度异质性，那么就需要进行亚组分析及敏感性分析等进一步探讨异质性的来源。

**2:**
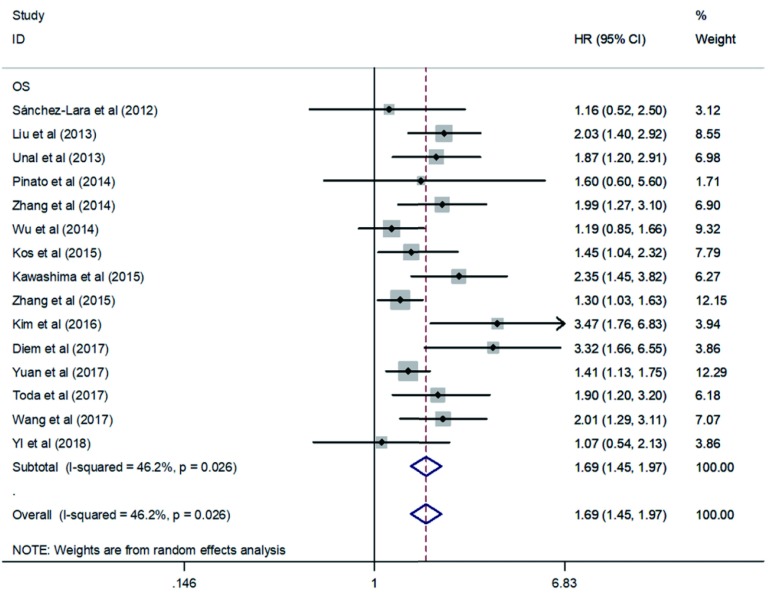
PLR与NSCLC患者OS关系的*Meta*分析森林图 Forest plot of the association between PLR and OS in patients with NSCLC. PLR: platelet lymphocyte ratio; OS: overall survival; NSCLC: non-small cell lung cancer

**3:**
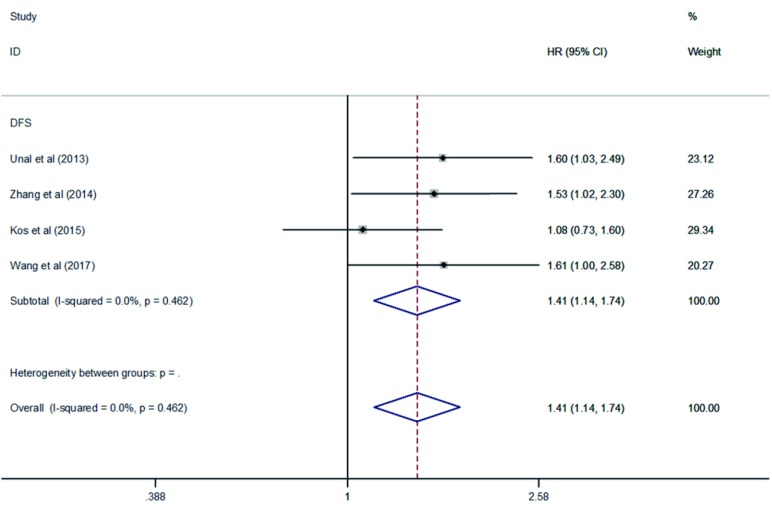
PLR与NSCLC患者DFS关系的*Meta*分析森林图 Forest plot of the association between PLR and DFS in patients with NSCLC. DFS: disease-free survival

### 2.4 亚组分析

为了进一步了解与NSCLC患者OS相关的研究文献之间异质性的来源，我们使用种族、PLR临界值、样本大小及治疗方式进行亚组分析（见图 4）。①当按人种划分进行亚组分析时，PLR增高，高加索人种（HR=1.75，95%CI: 1.29-2.38，P=0.0003，*I*^2^=25.1%，*P*异质性=0.254）及东亚人种（HR=1.68，95%CI: 1.39-2.01, *P* < 0.000, 01，*I*^2^=55.3%，*P*_异质性_=0.017）的患者预后不良（见图 4A）。②当按照PLR临界值> 162和PLR临界值≤162进行亚组分析时，结果显示合并的HR分别为1.78（95%CI: 1.44-2.21，*P* < 0.000, 01，*I*^2^=36%，*P*_异质性_=0.153）和1.61（95%CI: 1.27-2.03，*P* < 0.000, 1，*I*^2^=55.7%，*P*_异质性_=0.027）（见图 4B）。③当按样本大小进行亚组分析时，我们发现 > 202例患者的研究（HR=1.58，95%CI: 1.33-1.88，*P* < 0.000, 01，*I*^2^=41.9%，*P*_异质性_=0.099）和≤202例患者的研究（HR=1.85，95%CI: 1.39-2.48，*P* < 0.000, 1，*I*^2^=47.5%，*P*_异质性_=0.076）（见图 4C），从而得出结论，无论样本大小如何，PLR增高是NSCLC患者不利的预后因素。④当按治疗方式进行亚组分析时，我们发现手术治疗的4, 470例患者（HR=1.77，95%CI: 1.43-2.18，*P* < 0.000, 01，*I*^2^=51.4%，*P*_异质性_=0.044），非手术治疗的909例患者（HR=1.63，95%CI: 1.20-2.23，*P*=0.002，*I*^2^=56.2%，*P*_异质性_=0.044）（见图 4D），上述结果表明无论治疗方式如何，PLR增高仍然是NSCLC患者不利的预后因素。综上所述，亚组分析显示，按种族、样本大小、PLR临界值和治疗方法分组后，较高PLR组的OS明显低于较低PLR组的OS（*P* < 0.05）。

**4:**
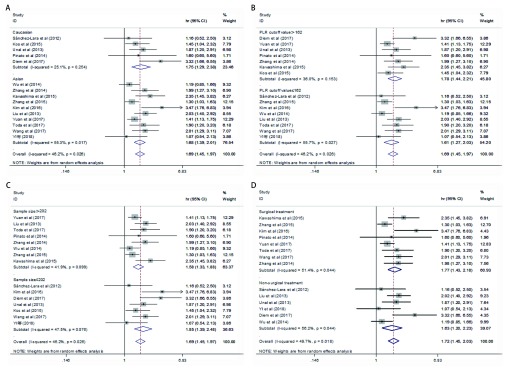
PLR与NSCLC患者OS关系的亚组分析森林图 Forest plot of the association between PLR and OS in patients with NSCLC by subgroup analyses

### 2.5 *Meta*回归分析

我们采用*Meta*回归分析的方法定量分析异质性的来源，当*P* < 0.1时可认为该因素可能为异质性的主要来源。进行单因素分析结果显示人种、样本大小、PLR临界值及治疗方式可能都不是研究间异质性的主要来源（表 2），多因素分析结果同样显示人种、样本大小、PLR临界值及治疗方式可能都不是研究间异质性的主要来源（表 3）。

**2:** *Meta*回归单因素分析 *Meta* regression single factor analysis

Variable	Standard deviation	*P*	95%CI
Ethnicity	0.1889	0.819	0.62-1.46
PLR cutoff value	0.1469	0.471	0.62-1.27
Sample size	0.1954	0.414	0.80-1.66
Treatment	0.2045	0.616	0.73-1.65

**3:** *Meta*回归多因素分析 *Meta* regression multivariate analysis

Variable	Standard deviation	*P*	95%CI
Ethnicity	0.3363	0.636	-0.59-0.93
PLR cutoff value	0.2245	0.318	-0.74-0.27
Sample size	0.2654	0.262	-0.28-0.91
Treatment	0.2172	0.729	-0.57-0.41

### 2.6 敏感性分析

我们进行了敏感性分析，目的是评估是否有个别研究影响了整体的分析结果。最终发现，依次去除任何一项研究，剩下的研究所合并的HR均在*Meta*分析中合并的HR的95%CI之内（图 5）。以上结果表明本*Meta*分析合并的HR具有良好的稳定性。

**5:**
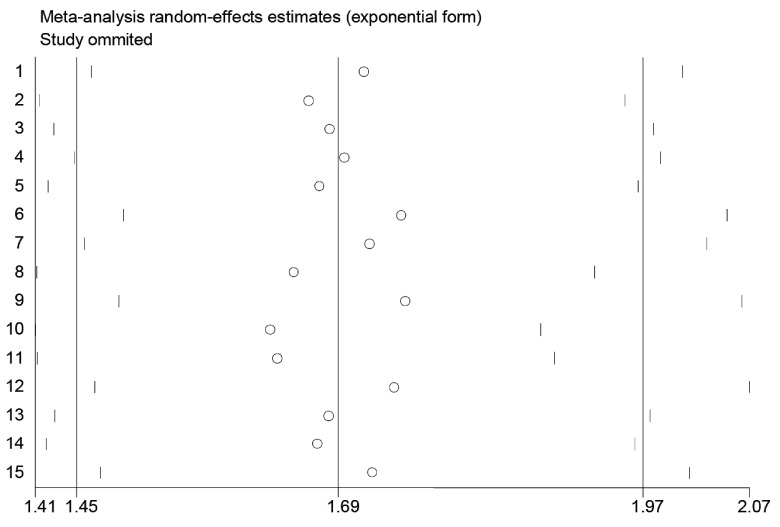
PLR与NSCLC患者OS关系的敏感性分析 Sensitivity analysis on the relationship between PLR and OS in NSCLC patients

### 2.7 发表偏倚

我们通过漏斗图（图 6）定性分析纳入的研究是否存在发表偏倚。图 6A、图 6B分别为OS的HR合并后的线性回归法（*Egger*法）和秩相关检验（*Begg*法）生成的漏斗图，图 6C、图 6D分别为DFS的HR合并后生成的漏斗图。图 6中可见稍有不对称，进一步进行定量分析，结果显示OS和DFS的HR合并后：Pr > |z|指标分别为0.235和0.308（*Begg*法）、Pr > |t|指标分别为0.053和0.256（*Egger*法），因此OS的HR合并后及DFS的HR合并后均不存在明显的发表偏倚。

**6:**
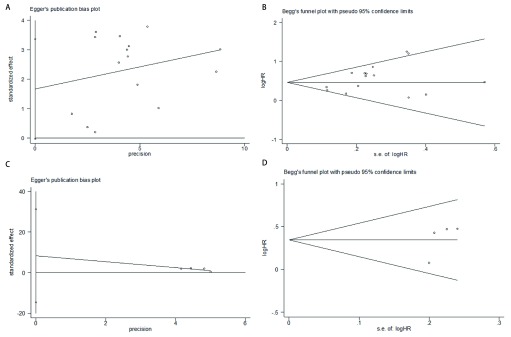
用于检测发表偏倚的漏斗图 Funnel plot for detecting publication bias

## 3 讨论

通过相关文献检索，我们发现国内目前分析较多的是PLR在肝细胞癌、结直肠癌、胃癌、宫颈癌及卵巢癌等恶性肿瘤患者中的预后价值，但是尚缺乏严格的分析PLR在NSCLC患者中的预后价值。根据此*Meta*分析结果显示，当PLR增高时会有相对降低的OS（HR=1.69，95%CI: 1.45-1.97，*I*^2^=46.2%，*P* < 0.000, 01，*P*_异质性_=0.026）和DFS（HR=1.41，95%CI: 1.14-1.74，*P*=0.001，*I*^2^=0%，*P*_异质性_=0.462）。根据*I*^2^值与*P*异质性值得出，纳入与NSCLC患者DFS相关的文献无明显异质性，但是纳入与NSCLC患者OS相关的研究文献之间存在中等程度异质性，其后就进行亚组分析及敏感性分析等方法进一步探讨异质性的来源。进行亚组分析时结果显示，当按患者的种族、样本大小、临界值及疾病分期等因素进行分析时，PLR增高仍然是NSCLC患者不利的预后因素。进行敏感性分析时显示本*Meta*分析合并的HR具有良好的稳定性。最后采用*Meta*回归分析的方法定量分析异质性的来源，单因素分析及多因素分析结果均显示人种、样本大小、PLR临界值及治疗方式可能都不是研究之间异质性的主要来源，其他如年龄、性别等方面的因素有可能为异质性的主要来源，但是由于缺少足够的数据因而无法进行分析。最后对发表偏倚进行定性及定量分析得出不存在明显的发表偏倚。在过去的几十年中，研究者们已经确定了许多关于NSCLC预后的因素，如遗传因素、患者状态、TNM分期等。同时，在前面的概述中我们也已经提到了目前多项研究显示，血小板、淋巴细胞、中性粒细胞、CRP以及GPS等，在多种癌症中都有重要的预后价值^[4-5]^。那么PLR对于癌症是否有重要的预后价值就需要进一步分析。目前数据显示PLR在肾细胞癌、食管癌、胃癌、肝癌以及结肠癌中有重要的预后价值^[6-10]^。那么PLR升高在NSCLC患者中是否有同样的预后价值，此*Meta*分析的结果显示是肯定的。

目前PLR在NSCLC患者中的预后相关机制仍未明确，但是我们检索到一些相关文献显示血小板及淋巴细胞在癌症进展中起重要的作用。例如已发表的研究显示，血小板增多症是NSCLC患者的独立的不利预后因素^[5]^。另一方面，也有研究^[30]^显示外周血淋巴细胞增多在NSCLC患者中是一个有利的预后因素。血小板聚集能够促进循环肿瘤细胞的粘附和包裹，这就使肿瘤细胞逃避免疫攻击的能力得到增强^[31]^。此外，活化的血小板能够释放多种血管内皮生长因子及多种细胞因子，从而增加肿瘤组织的血管生成最终促进其生长^[32-34]^。而淋巴细胞在抗肿瘤方面的作用现阶段研究非常多，其能通过再循环作用增加T细胞和B细胞与抗原和抗原提呈细胞接触的机会，从而产生免疫应答，有利于发挥各种免疫细胞和效应细胞的效应^[34]^。综上所述，PLR作为循环中血小板和淋巴细胞计数的比值，其升高则代表了血小板增多和（或）淋巴细胞减少，从而可能导致癌症患者病情的进展以及存活期的缩短。

此*Meta*分析的优点在于①立题相对较新，目前国内评估PLR对NSCLC患者的预后价值的文章非常少。②评估流程规范，严格按照相关指南进行开展，不管是文献检索还是质量评价，力求做到公正和客观。③结论具有一定的意义与价值，为NSCLC患者的预后提供了临床参考。同样此*Meta*分析还有一定的局限性：①根据先前进行文献检索时发现目前国内外做此方面研究大多是回顾性研究，因此本*Meta*分析纳入的大部分的研究都是回顾性研究，缺乏足够的前瞻性研究。②纳入分析的研究有限，各研究自身的数据水平无法判断且各组间的样本量不够平衡，同时缺少大样本的相关研究。③由于缺乏相关的数据，PLR与肿瘤相关的其他预后参数之间的相关性并未进行分析。④根据本*Meta*分析的结果，PLR作为NSCLC中预后相关的生物标记有一定临床意义，但同时中性粒细胞/淋巴细胞比值（neutrophil to lymphocyte ratio, NLR）、CRP等也为重要的预后影响因素之一，可能造成结果的可靠性降低。此*Meta*分析的结果显示高水平的PLR与NSCLC患者的OS和DFS的降低有着显著相关性，再加之PLR易于获得且成本较小，因此我们建议可将PLR用作NSCLC患者预后的生物标记，以帮助NSCLC患者进行个体化治疗方案的选择，然而PLR的临床应用仍需要未来通过更精良的设计和大规模、多平台、多中心的研究（最好是前瞻性研究）来验证。
